# Prominently enhanced luminescence from a continuous monolayer of transition metal dichalcogenide on all-dielectric metasurfaces

**DOI:** 10.1515/nanoph-2023-0672

**Published:** 2023-12-25

**Authors:** Masanobu Iwanaga, Xu Yang, Vasilios Karanikolas, Takashi Kuroda, Yoshiki Sakuma

**Affiliations:** National Institute for Materials Science (NIMS), 1-1 Namiki, Tsukuba 305-0044, Japan

**Keywords:** 2D materials, transition metal dichalcogenide, tungsten disulfide, all-dielectric metasurface, enhanced photoluminescence, resonance enhancement, coherent photon emitter

## Abstract

2D materials such as transition metal dichalcogenides (TMDCs) are a new class of atomic-layer materials possessing optical and electric properties that significantly depend on the number of layers. Electronic transitions can be manipulated in artificial resonant electromagnetic (EM) fields using metasurfaces and other designed nanostructures. Here, we demonstrate prominently resonant enhancement in the photoluminescence (PL) of atomic monolayer, WS_2_, doped with a small quantity of Mo. The excitonic PL showed a strong enhancement effect on a higher-order magnetic resonance of all-dielectric metasurfaces consisting of periodic arrays of Si nanopellets. The PL intensity witnessed a 300-fold enhancement compared to the reference PL intensity on a flat silicon dioxide (SiO_2_) layer, which suggests a drastic change in the dynamics of photoexcited states. Confocal PL microscopy and the analysis revealed that the single photons were coherently emitted from the TMDC monolayer on the metasurface. Furthermore, examining the PL lifetime in the ps and ns timescales clarified two exponential components at the prominent exciton PL: a short-time component decaying in 22 ps and a long-time component lasting over 10 ns. Therefore, we can infer that the radiative components were significantly activated in the TMDC monolayer on the metasurfaces in comparison to the reference monolayer on a flat SiO_2_ layer.

## 1 Introduction

One of the features of the transition metal dichalcogenides (TMDCs) is to constitute atomic-layer 2D materials and furthermore to comprises van der Waals layers. Their optical properties change drastically depending on the number of layers [1, 2]; in particular, the monolayers have a direct bandgap and exhibit strong excitonic luminescence. This feature greatly stimulated interest in fundamental studies on the luminescent properties. It was reported to date that the wavelengths of excitonic photoluminescence (PL) vary depending on the surrounding circumstance [3, 4], chemical [5, 6] or thermal [7] treatments, substrates [5, 8–10], and oxidation [11]. In addition to the surrounding circumstances, the reported results sometimes differed from others; for example, the substrate effect was reported such that PL intensity of WS_2_ monolayer was more than 10-fold larger on a sapphire substrate than an SiO_2_/Si substrate [8] whereas that was more than 5-fold weaker on a sapphire substrate than an SiO_2_/Si substrate [9]. Probably, the growth conditions substantially affected the results. Also, interpretations of the experimental results were sometimes inconsistent; for example, it was claimed that a TMDC monolayer suspended on a micrometer-diameter air hole emitted more intense PL than the monolayer on substrates [8] whereas it was reported that a skew monolayer showed larger PL intensity than a flat monolayer because a potential to excited carriers were induced [9]; in the former, monolayer curvature on a large air hole was neglected. Thus, there were sometimes confusions or inconsistency among the numerous studies in the early years of the TMDC study. However, we can summarize that the PL wavelengths coming from the excitons vary to some extent depending on the circumstances and treatments of the TMDC atomic layers. Additionally, dopant effect can alter the PL wavelengths [9, 12, 13], as was studied for many semiconductors.

To achieve a higher spontaneous emission (or PL) efficiency than that of the as-grown monolayers, TMDC monolayers were transferred to metallic [14–21] or dielectric [22–27] nanostructures. However, most of the results were primarily limited to PL-intensity enhancement factors (EFs) in the range of ten folds or less [14–18, 20–24, 26, 27]. A few exceptions reached more than a 100-fold enhancement for WSe_2_ [19], MoS_2_ [14], and WS_2_ [25]; the monolayers of WSe_2_ and MoS_2_ have advantage at the PL wavelengths longer than 650 nm because the wavelengths reduce the optical loss from the constituent noble metals forming the plasmonic nanostructures [14, 19]; in the case of WS_2_, the monolayer was transferred onto an Si_3_N_4_ grating and emitted directional PL via a diffraction mode, meaning that the geometric emission control mainly contributed to in the PL-intensity enhancement, whereas the claim of over 300-fold PL enhancement contradicts against the measured angle-resolved PL-intensity ratio indicating ∼25-fold [25]. Although the effective interplay of the nanostructures with the TMDC monolayers is considered to lead to obvious changes in the PL spectra, such results have rarely been reported, except for the plasmonic case for MoS_2_ [14] and dielectric nanophotonic structures for WSe_2_ [27, 28]. Trials were conducted to explore the prominent PL enhancement by transferring TMDC microstructures (primarily triangles) to the nanostructures. Recently, the wafer-scale growth of TMDC is in progress [29–32]; however, continuous atomic layers have not yet been employed for the PL-enhancing studies.

We point out the difference between TMDC-transferred nano- and microstructures. Most of the nanostructures have nano-size air gaps (∼100 nm); therefore, curvatures of monolayers on the air gaps are suppressed compared to the micrometer air hole (≥5 μm diameter) [8]. Thus, the potential trap effects due to the nano air gap are considered to be quite smaller than the micrometer hole, thereby being negligible.

Here, we show a clear change in the PL spectrum of a TMDC continuous atomic layer due to the resonance of all-dielectric metasurfaces composed of Si nanopellet (or short-height nanocolumn) array. Such a spectral change has not been explicitly demonstrated using any other dielectric nanostructures. Furthermore, we show a prominent 300-fold PL-intensity enhancement in comparison with the PL intensity on the reference flat SiO_2_ layer. The confocal PL image is shown to realize coherent single-mode emission, which obeys Poisson distribution and is direct evidence for single-photon emission. Such a direct imaging of planar quantum emitter has never been reported to our best knowledge. The enhanced PL was analyzed on the temporal profiles and consequently revealed that both fast (ps) and slow (ns) components are activated on the metasurfaces. The unusual PL responses are described in terms of exciton dynamics.

The all-dielectric metasurfaces in this study show highly enhanced fluorescence (FL) that exceeded 1000-fold magnification [33] and can be applied to FL biosensors targeting a wide range biomolecules from proteins to DNA [34–37]. A proof for single cell-free DNA detection was recently reported [38]. The metasurfaces are a counter part of plasmon–photon hybrid metasurfaces [39] with an extreme FL-enhancing capability [40–42]. In this article, we explore capability of the all-dielectric metasurfaces to enhance the PL of the TMDC atomic monolayer.

## 2 Results and discussion

**Figure 1:**
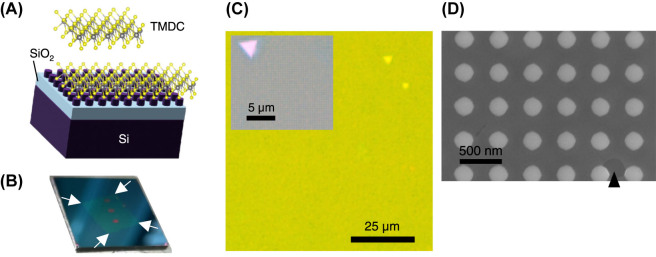
Structural overviews. (A) Schematic of TMDC monolayer (Gray: transition metal atoms. Yellow: sulfur atoms) and TMDC-transferred metasurface of Si-nanopellet array. (B) Photograph of an atomic-layer TMDC-transferred metasurface substrate of a 2-cm square. The corners of the TMDC are indicated by white arrows. Three metasurface areas are aligned along the centerline and under the TMDC layer. (C) Optical microscopy image of atomic-layer TMDC transferred on an all-dielectric metasurface. The atomic layer was primarily monolayer. The scale bar indicates 25 μm. Inset is a magnified view with a scale bar of 5 μm, where a triangle-shape bilayer is observed at the top left. (D) Top-view SEM image of the transferred TMDC monolayer onto the metasurface. The scale bar indicates 500 nm. The triangle indicates a broken hole in the TMDC layer.

A schematic illustration of a TMDC atomic monolayer and the TMDC-transferred metasurface composed of Si nanopellet array is shown in Figure 1A. The atomic layer was visualized using VESTA [43]. A photograph of the actual sample is shown in Figure 1B. The metasurface substrate used was a 2 cm × 2 cm square. The four white arrows indicate the corners of the continuous TMDC layer transferred onto the metasurface. The three metasurfaces are aligned vertically along the centerline and covered by the atomic layer. Each metasurface area was set to 1.2 mm × 0.6 mm dimensions. The growth and transfer methods are described in the Section 4.1 and 4.2, respectively.

### 2.1 Structures of TMDC-transferred metasurfaces

Figure 1C shows optical microscopy images of a TMDC-transferred metasurface. The TMDC film was nearly a monolayer that was grown under the condition described in the Methods section, whereas the μm-scale bilayers appear sparsely as triangle shapes. The black scale bar indicates 25 μm. The Inset shows a magnified view with a scale bar of 5 μm, which includes a triangle bilayer at the top left. A further magnified view is shown in Figure 1D, which was taken using a scanning-electron-microscopy (SEM) instrument (SU8230, Hitachi High-Tech, Tokyo, Japan); the scale bar indicates 500 nm. In the top-view SEM image, a hole in the TMDC layer indicated by a triangle is shown, which is in contrast to the transferred TMDC layer that covers approximately the entire top of Si-nanopellet array. Although the Si nanopellets were designed to be circular columns, they were approximated as regular octagons in the nanofabrication and had slight deviation from the perfect circles.

### 2.2 Optical resonances of the metasurface

**Figure 2:**
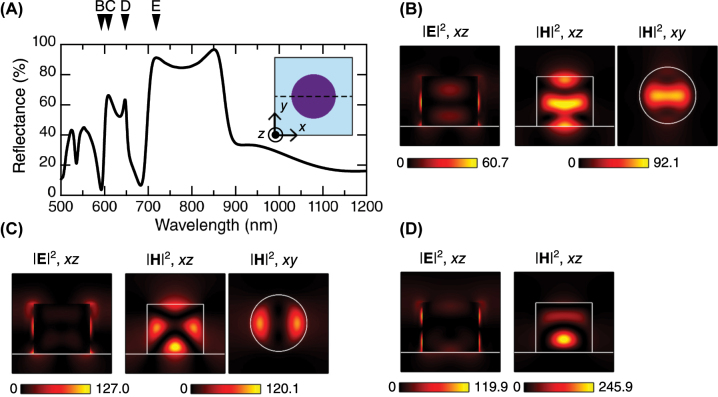
Optical resonances of the all-dielectric metasurface. (A) Computed reflectance spectrum of the metasurface consisting of a 400-nm-periodicity square array of Si nanopellets (or low-height nanocolumn) with a diameter of 228 nm. Triangles indicate resonant wavelengths B–E, corresponding to dip and peaks of the reflectance spectrum. Inset illustrates the unitcell in the *xy* plane on a *z* section; dashed line indicates *xz* section through the center of the Si nanopellet. (B)–(D) Resonant electric- and magnetic-field intensity distributions, |**E**|^2^ and |**H**|^2^, at the wavelengths indicated by the triangles in (A). The *xz* sections correspond to the dashed line in the inset of (A). The *xy* section is set at half the height of the Si nanopellet. Each intensity is represented by setting the incident field intensity to 1.0. See the Supplementary Material (Figure S1) for the |**E**|^2^ and |**H**|^2^ distributions at the condition of triangle E in (A).

To understand electromagnetic (EM) resonant modes of the all-dielectric metasurface itself, the numerically calculated reflectance spectrum is shown in Figure 2A. The metasurface was set in accordance with the actual sample (Figure 1D), such that the periodicity of array was 400 nm and the diameter of Si nanocolumn in the *xy* plane was 228 nm. Inset shows the unitcell at an *xy* section including the Si nanocolumn (purple). The optical resonances of the metasurface appear in the wavelength range below 900 nm. High reflectance peaks and deep dips exhibit prominent optical resonances. The four resonances relevant to this study are indicated by triangles B–E at the wavelengths of 592.5, 608.8, 646.9, and 718.1 nm, respectively, which are located near the PL wavelengths of the TMDC monolayer in this study.

The resonant EM-field distributions are shown in Figure 2B–D corresponding to the wavelengths B–D in Figure 2A, respectively. The incident EM fields were set to 1.0. In Figure 2B–D, the interfaces of the Si nanopellets and SiO_2_ layer are shown with white lines in the *xz* section, and the boundary of the Si nanopellets is shown with white lines and circles; the *xz* sections are set at the center of the Si nanopellets (dashed line in the inset of Figure 2A), and the *xy* sections are set at the half height of the Si nanopellets.

At the reflectance dip B, the magnetic fields are strongly localized in the Si nanopellet, forming a waveguide mode associated with a 92.1-fold enhancement in the field at the maximum. The electric field shows the greatest enhancement at the sidewall of the Si nanopellet, exceeding 60-fold in comparison to the incident electric field.

The magnetic-field distributions at the reflectance peak C are different from those at the dip B. Multipoles are observed in the Si nanopellet, exhibiting a higher-order magnetic resonance associated with more than a 120-fold enhancement in the field compared to that of the incident field. The electric fields are predominantly distributed at the outermost surface of the Si nanopellet; the intensity exhibits a 127-fold enhancement for that of the incident electric field. Thus, the enhanced electric fields available at the top of the Si nanopellet differ from those on the resonance in Figure 2B.

Although the reflectance peaks D and E are separated by a deep dip at 682.5 nm, their EM-field distributions are similar to each other. Figure 2D shows a strongly localized magnetic mode inside the Si nanopellets. More than 100-fold enhancements in the maximum intensities are observed for both electric and magnetic components, as compared to the incident field intensity. The enhanced electric fields are primarily distributed on the sidewalls of the Si nanopellets as shown in Figure 2D. Figure S1 in the Supplementary Material provides the field distributions at the reflectance peak E.

### 2.3 Enhanced PL spectrum and image

**Figure 3:**
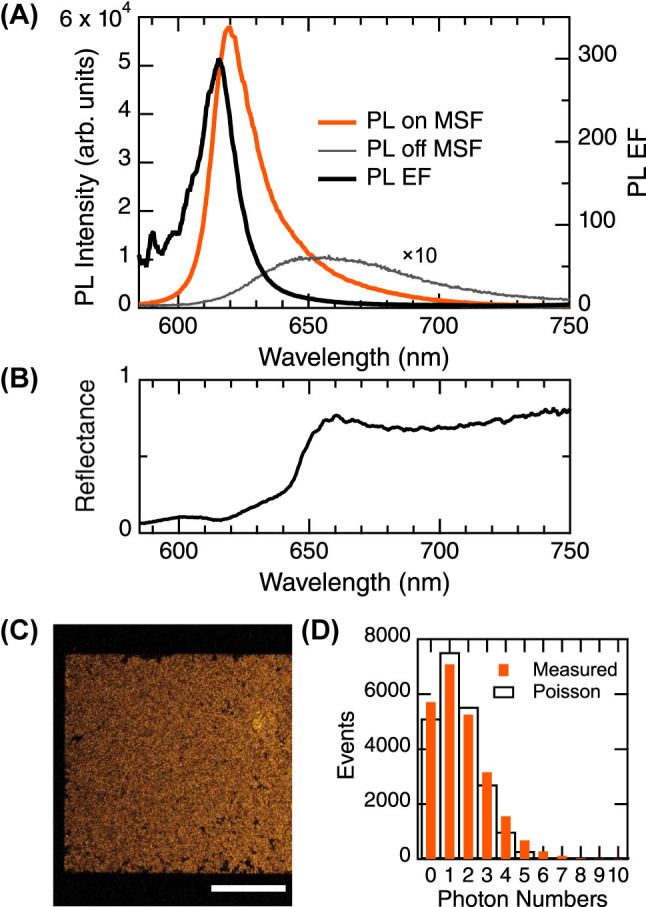
PL measurement on the metasurface (MSF). (A) Enhanced PL spectrum measured on the metasurface (orange). PL spectrum on the flat BOX layer (gray), which is 10-fold magnified for clarity. PL EF is plotted for the right axis (black solid). (B) Normalized reflectance spectrum of the TMDC-layer-transferred metasurface, measured at the normal incidence. (C) Confocal PL image, which is shown in orange-hot presentation. White scale bar indicates 200 μm. (D) Photon-number distribution (orange bars) evaluated from the confocal image in (B) and fitted using Poisson distribution (open black bars), which represents a quantum light effect, coherent single-mode emission [44].

Figure 3A shows the PL spectra measured under excitation at 532.0 nm. The PL spectra of the atomic layer on and off the metasurface are shown with orange and gray curves, respectively. The PL spectrum off the metasurface is magnified by 10-fold, for clarity. The PL EF spectrum, defined as the ratio of the PL spectra on and off the metasurface, is shown with a black curve plotted on the right axis. The peak value of the PL EF spectrum is as large as 300 at 614.5 nm. The peak of the PL spectrum on the metasurface is located at 619.4 nm whereas that off the metasurface is located at 650 nm. Thus, in addition to the large PL EF, a distinct spectral shape change occurs in the PL enhancement on the metasurface.

We note that the PL peak of WS_2_ monolayer on flat substrates can largely shift in a wavelength range from 620 to 670 nm, due to the substrate, transfer, and/or nm-scale strain [8, 9]. Thus, the PL peak at 650 nm off the metasurface in Figure 3A is a plausible result.

The enhanced PL-EF peak shows a good agreement with a reflectance dip at 616 nm in Figure 3B. The normalized reflectance spectrum was measured at the normal incidence on the TMDC-transferred metasurface, showing reflection of the coupled system of the TMDC monolayer and the metasurface. As a result, the reflectance is reduced below 650 nm, compared to the metasurface reflectance in Figure 2A, because of light absorption by the TMDC monolayer. Further, a small reflectance dip at 605 nm in Figure 3B is considered to originate from a reflectance peak C in the computation (Figure 2A). Thus, the measured peak of PL EF appeared near the resonance C of the metasurface. Note that, although there is another resonance of the reflectance peak D in Figure 2A, it does not contribute to the PL enhancement in 620–650 nm. Generally, prominent PL enhancement selectively appears at particular resonances [33, 40, 41]. The small dip at 616 nm is most likely a coupled resonance of the TMDC exciton with the optical resonance C in the metasurface. In short, the prominent PL enhancement at 614.5 nm takes place with help of a particular EM resonance in the metasurface.

Although many reports have addressed TMDC PL enhancement using artificially designed nanostructures [14–27], this type of resonant effect associated with definite changes of spectral shapes has not been reported, except for a previous report using plasmonic nanocavities [14]. This study moreover presents the largest value of PL EF for the TMDC monolayers, compared to those obtained by the dielectric nanostructured platforms previously reported.

We here refer to the optical properties of the as-grown TMDC atomic layers. The Raman-scattering and PL spectra are shown in the Supplementary Material (Figure S2). The Raman spectrum shows that the TMDC consists primarily of WS_2_ with a small quantity of Mo. The PL spectrum has a peak at 625 nm, representing *A* exciton of WS_2_. As we mentioned the previous reports [8, 9], transfer of TMDC film often resulted in PL-peak shift, which was observed in this study as well. As shown in Figure 3A, the TMDC transferred onto the SiO_2_ layer has a PL peak at 650 nm.

High-spatial-resolution PL images were acquired using an upright confocal FL microscope (Stellaris 5, Leica Microsystems, Wetzlar, Germany). One of the PL images is shown in Figure 3C, where the pseudo-color representation is orange-hot and the white scale bar indicates 200 μm. Rectangular colored area corresponds to the metasurface. Evidently, the PL becomes very dark outside the metasurface though the TMDC atomic layer covers the outside as well. In the confocal image, the PL intensity outside the metasurface is indistinguishable from the zero level in the measurement. The confocal PL image was acquired in a photon-counting manner; therefore, the PL intensity was quantified by examining the number of emitted photons.

Setting an analyzing region on the metasurface (Figure S3), the distribution of photon numbers was obtained, as shown in Figure 3D. The vertical axis represents number of photon-emission events. The photon numbers (orange bars) are small and primarily limited to five or fewer, including 14.4 % zero-photon and 29.7 % one-photon events. The distribution was fitted using the Poisson distribution and reproduced fairly well, being found to have mean photon number of 1.47. Small deviation is probably due to structural imperfection of the TMDC monolayer: the triangular bilayers and the broken holes as seen in Figure 1C and D, respectively. The fitted Poisson distribution is plotted in Figure 3D using open black bars. The photon distribution obeying Poisson distribution represents coherent single-mode photon emission [44], which is direct evidence for a quantum light emitter. It thus turns out that the confocal PL image (Figure 3C) directly visualizes a TMDC quantum light emitter. As a corollary of the coherent single-mode emission, the degree of second-order coherence *g* ^(2)^ is derived to be unity, irrespective of the mean photon numbers [44]. Note that the confocal PL imaging is more informative than the conventional *g* ^(2)^ measurement, which is reported frequently as experimental evidence of single-photon emission, and completes in much shorter time of tens of seconds than the time-consuming *g* ^(2)^ measurement.

### 2.4 Decay-time analysis of enhanced PL

**Figure 4:**
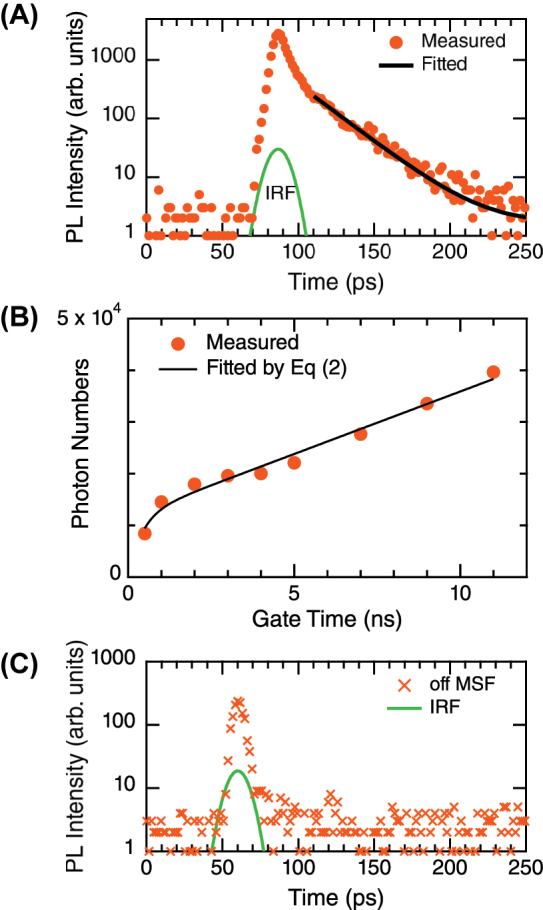
Temporal profiles of measured PL. (A) And (B) fast and slow components in ps and ns ranges, respectively. Measured data are shown with orange closed circles and fitted curves with solid curves in both (A) and (B). The fitting exponential functions, Equations (1) and (2) for (A) and (B), respectively, are described in the text. In (A), the PL intensity is shown on the logarithmic scale, and the temporal profile of ps excitation laser pulse is drawn with a green dotted curve for reference. (C) PL profile measured off the metasurface (orange crosses). IRF to the ps laser pulse (or the measured temporal profile) is also shown with green curves in (A) and (C).

Figure 4A shows the PL decay profile in a ps range. The PL was excited using laser pulses with 2-ps duration and 540-nm wavelength and was detected around the PL-peak wavelengths. The instrumental response function (IRF) of the laser pulses in the setup used is shown with a green curve, and it has a full width at the half maximum of 10 ps. The initial response of the PL decay curve follows the IRF. The ultrafast response suggests that photocarriers are immediately consumed via a nonradiative process. In the time range later than 15 ps from the peak of the laser pulse, the measured data (orange closed circles) are fitted using an exponential curve (black solid curve) such that

(1) $$y=y_{0}+C_{0}\exp\left[-\left(x-x_{0}\right)/\tau_{s}\right]$$

where *C*_0_ is a proportional constant, *x*_0_ is time offset, and *τ*_*s*_ is decay time in the ps range. The fitting results as shown in Figure 4A revealed the value of *τ*_*s*_ = 22.3 ± 0.3 ps. The PL lifetime under the excitation condition was approximately 10 ps in a previous time-resolved study [45]. It was also reported that a WS_2_ monolayer grown on SiO_2_/Si had the decay time of 11.4 ps and a partially oxidized WS_2_ monolayer had that of 31.5 ps  [11]. Thus, the measured *τ*_*s*_ lies in a plausible range.

To analyze the temporal profile of PL in a ns range, in Figure 4B, the time-integrated PL intensity over [0, *T*] (orange dot) is plotted for the gate time *T* of a photon-counting detector. The growing temporal profile *I*(*T*) was defined, using an integrated double-exponential function, such that

(2) $$\begin{array}{ll} I\left(T\right) & =\overset{T}{\underset{0}{\int }}\left[C\exp\left(-t/\tau_{s}\right)+D\exp\left(-t/\tau_{L}\right)\right]dt\\ & \approx C\tau_{s}\left[1-\exp\left(-T/\tau_{s}\right)\right]+DT \end{array}$$

and fitted the measured data (solid black curve) where a relation *τ*_*L*_ ≫ *T* was assumed to derive Equation (2). *C*, *D*, and *τ*_*s*_ are the fitting parameters and the fitted curve is shown with a black curve, which well reproduces the PL-growing profile. The short lifetime *τ*_*s*_ was evaluated to be 0.41 ± 0.16 ns; consequently, for gate time longer than 5 ns, the profile *I*(*T*) dominantly depends on *T* and shows an approximately linear increase in Figure 4B. This result indicates that the *τ*_*L*_ is sufficiently longer than the maximum gate time of 11 ns. In Figure 4B, the total PL intensity coming from the long lifetime component, *Dτ*_*L*_, is larger than that from the short component, *Cτ*_*s*_, whereas the ratio of populations in the fast and slow components, i.e., *C* : *D* is determined to be 11.8 : 1 using by Equation (2). These results imply that there are two possible excitonic transitions in the TMDC monolayer. A theoretical study on the exciton bands of monolayer MoS_2_ showed that the *A* excitons have *K*–*K* direct and *K*–Γ intervalley transitions at approximately the same energy [46], where *K* and Γ denote the high-symmetry points in the Brillouin zone. Because WS_2_ is considered to have similar exciton bands to those of MoS_2_, the two excitonic transitions are attributed to the two observed PL components.

We mention that the *τ*_*s*_ is approximately one-order longer than that found in the ps range. This is because the temporal resolution in Figure 4B is limited by the minimum gate time of 0.5 ns. Thus, the evaluated *τ*_*s*_ in the ns range suggests that *τ*_*s*_ is shorter than 0.4 ns and does not imply inconsistency with the analysis in the ps range (Figure 4A). The precise evaluation of *τ*_*s*_ is conducted in the ps range.

The PL decay was also measured off the metasurface and then, the TMDC layer was on placed the SiO_2_ layer. These data are shown with orange crosses in Figure 4C. The ps-laser-pulse IRF is shown together with a green curve, being in agreement with the PL profile. The PL decay was also fitted by an exponential curve (not shown here) and the decay time was 4.7 ± 0.1 ps. These results indicates that the PL decays together with the incident laser pulse and strongly suggests the PL predominantly decays within 5 ps via ultrafast nonradiative process(es) because the PL intensity is heavily reduced in comparison with that of the TMDC monolayer on the metasurface. In other words, the photocarriers in the TMDC film off the metasurface are immediately consumed through the nonradiative path(s).

### 2.5 Resonantly enhanced PL processes

**Figure 5:**
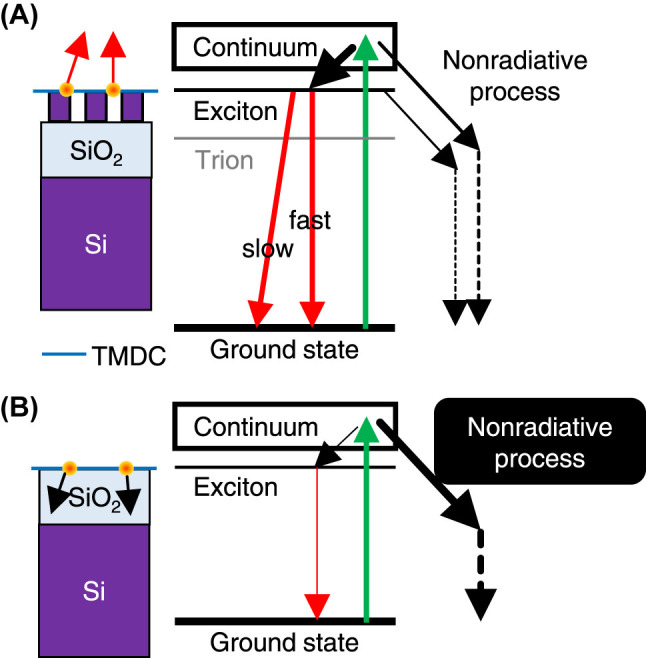
Schematic of the PL dynamics: (A) and (B) TMDC monolayers (blue) on and off the metasurface, respectively. Structural schematics are drawn on the left side; excitons are represented with orange rounds. Energy diagrams are depicted on the right side. Green arrows represent photoexcitation. Solid red and black arrows denote radiative and excitation transfer processes, respectively.Dashed black arrows indicate nonradiative decay. See more details in the text.

Figure 5A and B shows schematics of the PL dynamics on and off the metasurface, respectively. Blue lines denote the TMDC monolayers. Schematics of the structures and energy diagrams are drawn on the left and right sides, respectively. On the metasurface (Figure 5A), photoexcited carriers are considered to have two major relaxation paths based on the analysis of PL decay time: one is the nonradiative process(es) and the other is the excitonic transitions. Green arrows indicate excitation using the laser light. Excited-state transfer is represented with solid black arrows. Radiative and nonradiative transitions are shown with red and dashed black arrows, respectively. The thickness of the arrows represents the probability of each process; the thicker arrows denote higher probabilities. As shown in the temporal profiles (Figure 4), the excitonic transitions have fast and slow components. However, these components are indistinguishable in the PL spectrum, because the transitions occur at approximately the same energy [46]. In Figure 5A, the vertical red arrow denotes the direct excitonic transition and the oblique red arrow does the indirect (or intervalley) transition.

In contrast, off the metasurface (Figure 5B), the PL dynamics are attributable to dominant nonradiative process(es), indicating that nearly all the photocarriers are consumed without emitting PL. A damping oscillator model [47] clarified that the excitation transfer on the top of dielectric becomes drastically fast, when the distance between the excited state and the surface of dielectric is close within a few nm [40]; the transfer rate exceeds 10^5^ at the distance of 0.1 nm, is more than 10^3^ at 0.5 nm, and is in the one-digit order at 5 nm and more. Although the excited states in the TMDC are not simple isolated oscillators, it is probable that the excitation transfer from the TMDC layer to the SiO_2_ layer takes place in a similar manner, as indicated by the black arrows in Figure 5B.

### 2.6 PL EF

The PL EF in the experimental configuration is expressed as [33, 40, 48]

(3) $$EF=\frac{N_{m}}{N_{0}}\times \frac{\eta_{m}}{\eta_{0}}\times \frac{\gamma_{m}\left(k\right)}{\gamma_{0}\left(k\right)}$$

where the subscripts *m* and 0 denote on and off metasurface, respectively. *N* is the photoexcited populations, *η* is the quantum yield, and *γ*(**k**) is the radiative decay rate dependent on outgoing wavevector **k**. In particular, *η* = *γ*/(*γ* + *γ*_NR_) where *γ*_NR_ means nonradiative decay rate. The ratio *γ*_*m*_/*γ*_0_ is often referred to as the Purcell factor [49].

The electric-field intensity at the excitation wavelength (Figure S4) shows that the TMDC monolayer was excited efficiently by a maximum of 5.3-fold, which gave the ratio *N*_*m*_/*N*_0_. Other terms in Equation (3) were based on the PL processes and Purcell effect.

The Purcell effect was often discussed with PL enhancement of the TMDC atomic layers [20, 27]. In a simple scheme describing two-level systems in optical resonators, the spontaneous emission is expedited through the resonant electric fields. We numerically explored the Purcell effect in this configuration using the all-dielectric metasurfaces; the details are noted in the Section 4.6. In case of *z* polarization, the total Purcell factor was as large as 180 at the maximum, whereas, for the in-plane (i.e., *x* or *y*) polarization, it was approximately 2 in the range of 600–650 nm (Figure S5). The total Purcell factor contains both radiative and nonradiative effects. The *A* exciton in the TMDC is limited to the in-plane polarization [50]. Further, a radiative effect is observed in the PL measurement. Overall, the factor *γ*_*m*_/*γ*_0_ is at most 2 in Equation (3) and the other contribution comes from the ratio *η*_*m*_/*η*_0_, which is estimated to be 30 or less. As illustrated in Figure 5, the change in exciton relaxation dynamics is a major factor yielding the large PL EF (Figure 3A).

## 3 Conclusions

The experimental investigation of a coupled system composed of all-dielectric metasurfaces and a large-area continuous TMDC monolayer revealed that the exciton PL spectrum was 300-fold enhanced at the maximum for the reference PL spectrum. This enhancement is the most significant as compared to those in the previous studies using dielectric nanostructures [22–27]. The analysis for the confocal PL image revealed that coherent single-mode photon emissions on the metasurface. The single mode substantially includes single-photon emission [44]. Thus, this coupled system was found to function as a single-photon generator at room temperature. The enhanced PL was also measured and analyzed with respect to the decay time, and the fast and slow radiative components were identified in the ps and ns ranges, respectively. The two components were accountable for direct and intervalley transitions of the *A* excitons. Overall, the highly enhanced PL was achieved based on a multiple effect of efficient excitation, activated radiative excitonic processes, and Purcell effect in this resonance-tuned coupled system.

## 4 Experimental section

### 4.1 TMDC growth

Atomic-layer WS_2_ films were grown on *c*-plane sapphire substrates via cold-wall chemical vapor deposition at 800 °C and 50 Torr for 60 min. H_2_S and WOCl_4_ were used as sulfur and tungsten precursors, respectively. The flow rate of H_2_S was 1 sccm. The feed rate of N_2_ carrier gas through the WOCl_4_ canister was 300 sccm, and the temperature and pressure of the canister were maintained at 45 °C and 760 Torr, respectively. The total N_2_ flow rate in the reactor was 2500 sccm. Additionally, 0.1 sccm O_2_ was injected during the deposition of WS_2_. Dragontrail glass serving as a catalyst reservoir was placed upstream of the sapphire substrate during growth, providing Na and K catalysts and promoting the lateral growth of WS_2_ with enlarged grain size.

Due to the previous usage of the chamber for the growth of MoS_2_ monolayers, a trace amount of Mo was found in the Raman spectrum of the as-grown film (Figure S2); however, because the ratio of Mo is estimated to be small (10 % or less) [13] and plays a minor role in the optical properties, we simply refer to the TMDC as WS_2_.

### 4.2 Transfer process onto metasurfaces

The as-grown monolayer WS_2_ films were transferred from the sapphire onto the metasurface. The procedure is schematically illustrated in Figure S6. Before the delamination, the WS_2_/sapphire sample was coated with polymethyl methacrylate (PMMA) to support and protect the ultrathin WS_2_. Subsequently, the PMMA/WS_2_ stack was peeled off from the sapphire surface by allowing deionized water to penetrate the interface between the sapphire and WS_2_. Finally, the sample of PMMA/WS_2_ on the above-mentioned metasurface was immersed in acetone at 50 °C for 60 min to remove the PMMA and then cleaned in isopropanol, followed by drying with a nitrogen blow.

After the transfer, rapid temperature annealing was performed, which started at room temperature; the temperature was raised to 300 °C and kept for 10 min under N_2_ gas flow at 120 sccm; subsequently, the sample was cooled down to room temperature under N_2_ gas flow. This annealing process improved the PL intensity a few times at the maximum.

### 4.3 Nanofabrication of metasurfaces

Metasurfaces were fabricated based on the Si-on-insulator wafers; the top Si layer was 200 nm thick, the middle SiO_2_ layer was 375 nm, and the base Si wafer was 675 μm. The top Si layer was crystalline with the (100) surface and had a p-type dopant. The nanofabrication procedures using electron-beam lithography and selective dry etching of the Si layer have been described elsewhere in detail [34, 42].

### 4.4 Optical measurement

The PL of the TMDC on the metasurfaces was measured at room temperature using a micro-PL setup for the spectrum acquisition and a confocal microscope for the spectrally resolved PL images. In the micro-PL setup, single-mode continuous-wave laser light of emission wavelength 532.0 nm (Action532Q-0050, AOTK, China) was focused on the TMDC monolayer on the metasurface using a 50× objective lens with a numerical aperture (NA) 0.55 (M Plan Apo, Mitsutoyo, Kawasaki, Japan) and a working distance of 13.0 mm. The PL from the TMDC monolayer was collected using the objective lens. The laser power was set to 0.5 mW or less at the entrance of the objective lens. The collected PL was passed through a monochromator (ISO-160, Teledyne Princeton Instruments, Trenton, NJ, USA), and the PL spectra were acquired using a cooling CCD camera (ProEM1024HS, Teledyne Princeton Instruments). The laser power was set to 0.5 mW or less at the entrance of the objective lens. When comparing the PL intensities, we kept the laser power to be a constant value.

Normal reflectance in Figure 3B was measured using the setup for the micro-PL measurement above. A 10× objective lens of NA 0.28 (M Plan Apo, Mitsutoyo) was used. Light source was a halogen lamp and two apertures were inserted at the entrance of the objective lens and an imaging lens (MT-L, Mitsutoyo). The apertures were set to be narrow and the incident angle of incoming beam was limited to be less than 0.3°, ensuring the normal incidence practically. Reference signals were measured using a calibrated Al mirror. Thus, we quantitatively evaluated reflectance of the TMDC–metasurface coupled system.

For the confocal PL image shown in Figure 3C, the excitation wavelength was set to 514.0 nm and a 10× objective lens of NA 0.32 (HC PL Fluotar, Leica Microsystems) was used. The lateral resolution was 819.2 nm. The PL growth in the ns range as shown in Figure 4B was measured by varying the gate width of the photon-counting detector in the confocal microscope.

PL decay-time measurements were conducted using a 76-MHz-repetition pulsed laser (MIRA-OPO-X ps, Coherent, Santa Clara, CA, USA) with an emission wavelength of 540 nm and a pulse width of 2 ps. The pulsed laser light was focused using a 50× objective lens with NA 0.8 and a working distance of 1.0 mm (MPFLN50X, Olympus, Tokyo, Japan). The laser power injected in the objective lens was 20 and 100 μW for the TMDC monolayer on and off the metasurface, respectively. The PL was collected using the same objective lens. The detection wavelengths were set to 615–620 and 640–650 nm on and off the metasurface, respectively, in accordance with the PL peaks (Figure 3A). The PL in the ps range was acquired using a synchronously scanning streak camera (C5680, Hamamatsu Photonics, Hamamatsu, Japan), attached to a monochromator (250is, Chromex, Albuquerque, NM, USA). The IRF with a full width at half maximum of 10 ps (Figure 4A and C) was determined by measuring the time traces of the excitation pulses.

### 4.5 Computation of reflectance spectra and EM-field distributions

The reflectance spectrum in Figure 2 and the emittance spectrum in Figure 3 were computed based on the rigorous coupled-wave analysis (RCWA) method [51], which was combined with a scattering-matrix algorithm [52] for numerical stability. The RCWA code was implemented on a supercomputer. The EM-field distributions in Figure 2 were output using the RCWA method as well. A material parameter, that is, the permittivity of crystalline Si constituting the metasurface were taken from literature [53]. The permittivities of air and SiO_2_ in the wavelength range of interest were set to their representative values of 1.00054 and 2.1316, respectively.

### 4.6 Numerical evaluation of purcell factor

To evaluate the Purcell factors in Equation (3), the excitons in the WS_2_ layer were approximated as two-level energy quantum systems. Their Purcell factors were calculated using the macroscopic quantum electrodynamic theory. The excitation created by an electric dipole source is connected in quantum mechanical terms with the relaxation of the exciton. In the real terms, the relaxation of the exciton was obtained by solving the Maxwell equations for a point dipole excitation in a complicated environment, which includes the EM response of the involved materials. A commercial finite-difference time-domain (FDTD) software (Ansys, Canonsburg, PA, USA) was used to solve the Maxwell equations. The optical responses of the different materials were determined from their experimentally measured dielectric permittivities [53].

Physically, the Purcell factor, Γ(*r*, *ω*), gave the enhancement or inhibition of the relaxation rate of the exciton when the WS_2_ monolayer was placed in the nanostructured environment, compared to the reference relaxation value of the WS_2_ monolayer. A reference value was used for the case where the WS_2_ layer was placed in a homogeneous media. In the reference media, the WS_2_ excitons followed an exponential relaxation, exp(−*t*/*τ*_ref_), from the excited to the ground state, where *τ*_ref_ is the reference lifetime. In the presence of the nanostructured environment, the relaxation was accelerated by the Purcell factor value to exp(−Γ*t*/*τ*_ref_). The Purcell factor is given by Γ = *τ*_ref_/*τ*_NS_, where *τ*_NS_ is the lifetime of the exciton in the nanostructured environment. The factor Γ can also be expressed as Γ = *γ*_*m*_/*γ*_0_, which is a factor on the right-hand side of Equation (3).

## Supplementary Material

Supplementary Material is available online. Figure S1: EM-field distributions at the reflectance peak E in Figure 2A. Figure S2: Raman-scattering and PL spectra of the as-grown TMDC atomic layer. Figure S3: Confocal PL image with the analyzing box. Figure S4: EM-field distributions of the metasurface at 532 nm. Figure S5: Numerically evaluated Purcell factors. Figure S6: Illustrative description for the transfer procedure.

## Supplementary Material

Supplementary Material Details

## References

[1] Splendiani A., Sun L., Zhang Y. (2010). Emerging photoluminescence in monolayer MoS_2_. *Nano Lett*..

[2] Mak K. F., Lee C., Hone J., Shan J., Heinz T. F. (2010). Atomically thin MoS_2_: a new direct-gap semiconductor. *Phys. Rev. Lett.*.

[3] Fu Y., He D., He J. (2019). Effect of dielectric environment on excitonic dynamics in monolayer WS_2_. *Adv. Mater. Interfaces*.

[4] Tanoh A. O. A., Alexander-Webber J., Xiao J. (2019). Enhancing photoluminescence and mobilities in WS_2_ monolayers with oleic acid ligands. *Nano Lett*..

[5] McCreary K. M., Hanbicki A. T., Singh S. (2016). The effect of preparation conditions on Raman and photoluminescence of monolayer WS_2_. *Sci. Rep.*.

[6] Aihara T., Wang R., Yang X., Sakuma Y., Okano A. O., Ikezawa M. (2022). Observation of strain relaxing in nanoscale WS_2_ monolayers grown on SiO_2_/Si by organic solvent treatment. *Jpn. J. Appl. Phys.*.

[7] Hua X., Axenie T., Goldaraz M. N. (2022). Improving the optical quality of MoSe_2_ and WS_2_ monolayers with complete *h*-BN encapsulation by high-temperature annealing. *ACS Appl. Mater. Interfaces*.

[8] Yu Y., Yu Y., Xu C. (2016). Engineering substrate interactions for high luminescence efficiency of transition-metal dichalcogenide monolayers. *Adv. Funct. Mater.*.

[9] Cong C., Shang J., Wang Y., Yu T. (2018). Optical properties of 2D semiconductor WS_2_. *Adv. Opt. Mater.*.

[10] Zhang F., Erb C., Runkle L., Zhang X., Alem N. (2018). Etchant-free transfer of 2D nanostructures. *Nanotechnology*.

[11] Luo Z., Zheng W., Luo N. (2022). Photoluminescence lightening: extraordinary oxygen modulated dynamics in WS_2_ monolayers. *Nano Lett*..

[12] Tongay S., Fan W., Kang J. (2014). Tuning interlayer coupling in large-area heterostructures with CVD-grown MoS_2_ and WS_2_ monolayers. *Nano Lett*..

[13] An X., Zhao W., Yu Y. (2022). Resonance Raman scattering on graded-composition W_x_Mo_1−x_S_2_ alloy with tunable excitons. *Appl. Phys. Lett.*.

[14] Huang J., Akselrod G. M., Ming T., Kong J., Mikkelsen M. H. (2018). Tailored emission spectrum of 2D semiconductors using plasmonic nanocavities. *ACS Photonics*.

[15] Butun S., Tongay S., Aydin K. (2015). Enhanced light emission from large-area monolayer MoS_2_ using plasmonic nanodisc arrays. *Nano Lett*..

[16] Akselrod G. M., Ming T., Argyropoulos C. (2015). Leveraging nanocavity harmonics for control of optical processes in 2d semiconductors. *Nano Lett*..

[17] Kern J., Trügler A., Niehues I. (2015). Nanoantenna-enhanced light–matter interaction in atomically thin WS_2_. *ACS Photonics*.

[18] Wang S., Li S., Chervy T. (2016). Coherent coupling of WS_2_ monolayers with metallic photonic nanostructures at room temperature. *Nano Lett*..

[19] Wang Z., Dong Z., Gu Y. (2016). Giant photoluminescence enhancement in tungsten-diselenide–gold plasmonic hybrid structures. *Nat. Commun.*.

[20] Indukuri S. R. K., Frydendahl C., Bar-David J., Mazurski N., Levy U. (2020). WS_2_ monolayers coupled to hyperbolic metamaterial nanoantennas: broad implications for light–matter-interaction applications. *ACS Appl. Nano Mater.*.

[21] Du B., Li Y., Jiang M. (2022). Polarization-dependent purcell enhancement on a two-dimensional h-BN/WS_2_ light emitter with a dielectric plasmonic nanocavity. *Nano Lett*..

[22] Chen H., Nanz S., Abass A. (2017). Enhanced directional emission from monolayer WSe_2_ integrated onto a multiresonant silicon-based photonic structure. *ACS Photonics*.

[23] Cihan A. F., Curto A. G., Raza S., Kik P. G., Brongersma M. L. (2018). Silicon mie resonators for highly directional light emission from monolayer MoS_2_. *Nat. Photonics*.

[24] Bucher T., Vaskin A., Mupparapu R. (2019). Tailoring photoluminescence from MoS_2_ monolayers by Mie-resonant metasurfaces. *ACS Photonics*.

[25] Li H., Wang J., Ma Y. (2020). Enhanced directional emission of monolayer tungsten disulfide (WS_2_) with robust linear polarization via one-dimensional photonic crystal (PhC) slab. *Nanophotonics*.

[26] Yan J., Zheng Z., Lou Z., Li J., Mao B., Li B. (2020). Enhancement of exciton emission in WS_2_ based on the Kerker effect from the mode engineering of individual Si nanostripes. *Nanoscale Horiz.*.

[27] Azzam S. I., Parto K., Moody G. (2023). Purcell enhancement and polarization control of single-photon emitters in monolayer WSe_2_ using dielectric nanoantennas. *Nanophotonics*.

[28] Wu S., Buckley S., Schaibley J. R. (2015). Monolayer semiconductor nanocavity lasers with ultralow thresholds. *Nature*.

[29] Yang P., Zou X., Zhang Z. (2018). Batch production of 6-inch uniform monolayer molybdenum disulfide catalyzed by sodium in glass. *Nat. Commun.*.

[30] Yang X., Li S., Ikeda N., Sakuma Y. (2022). Oxide scale sublimation chemical vapor deposition for controllable growth of monolayer MoS_2_ crystals. *Small Methods*.

[31] Huang C.-C., Wang H., Cao Y. (2022). Facilitating uniform large-scale MoS_2_, WS_2_ monolayers, and their heterostructures through van der Waals epitaxy. *ACS Appl. Mater. Interfaces*.

[32] Yang X., Li S., Ikeda N., Ohtake A., Sakuma Y. (2023). Scalable growth of atomically thin MoS_2_ layers in a conventional MOCVD system using molybdenum dichloride dioxide as the molybdenum source. *Appl. Surf. Sci.*.

[33] Iwanaga M. (2018). All-dielectric metasurfaces with high-fluorescence-enhancing capability. *Appl. Sci.*.

[34] Iwanaga M. (2020). All-dielectric metasurface fluorescence biosensors for high-sensitivity antibody/antigen detection. *ACS Nano*.

[35] Iwanaga M. (2021). High-sensitivity high-throughput detection of nucleic-acid targets on metasurface fluorescence biosensors. *Biosensors*.

[36] Iwanaga M., Tangkawsakul W. (2022). Two-way detection of COVID-19 spike protein and antibody using all-dielectric metasurface fluorescence sensors. *Biosensors*.

[37] Iwanaga M. (2022). Rapid detection of attomolar SARS-CoV-2 nucleic acids in all-dielectric metasurface biosensors. *Biosensors*.

[38] Iwanaga M., Hironaka T., Ikeda N., Sugasawa T., Takekoshi K. (2023). Metasurface biosensors enabling single-molecule sensing of cell-free DNA. *Nano Lett*..

[39] Iwanaga M., Choi B. (2015). Heteroplasmon hybridization in stacked complementary plasmo–photonic crystals. *Nano Lett*..

[40] Choi B., Iwanaga M., Miyazaki H. T., Sugimoto Y., Ohtake A., Sakoda K. (2015). Overcoming metal-induced fluorescence quenching on plasmo-photonic metasurfaces coated by a self-assembled monolayer. *Chem. Commun.*.

[41] Iwanaga M., Choi B., Miyazaki H. T., Sugimoto Y. (2016). The artificial control of enhanced optical processes in fluorescent molecules on high-emittance metasurfaces. *Nanoscale*.

[42] Iwanaga M. (2021). Highly sensitive wide-range target fluorescence biosensors of high-emittance metasurfaces. *Biosens. Bioelectron.*.

[43] Momma K., Izumi F. (2011). Vesta 3 for three-dimensional visualization of crystal, volumetric and morphology data. *J. Appl. Crystallogr.*.

[44] Loudon R. (2000). *The Quantum Theory of Light*.

[45] Kuroda T., Hoshi Y., Masubuchi S. (2020). Dark-state impact on the exciton recombination of WS_2_ monolayers as revealed by multi-timescale pump-probe spectroscopy. *Phys. Rev. B*.

[46] Wu F., Qu F., MacDonald A. H. (2015). Exciton band structure of monolayer MoS_2_. *Phys. Rev. B*.

[47] Chance R. R., Prock A., Silbey R. (1978). Molecular fluorescence and energy transfer near interfaces. *Adv. Chem. Phys.*.

[48] Iwanaga M. (2016). *Plasmonic Resonators: Fundamentals, Advances, and Applications*.

[49] Purcell E. M. (1946). Spontaneous emission probabilities at radio frequencies. *Phys. Rev.*.

[50] Munkhbat B., Wróbel P., Antosiewicz T. J., Shegai T. O. (2022). Optical constants of several multilayer transition metal dichalcogenides measured by spectroscopic ellipsometry in the 300–1700 nm range: high index, anisotropy, and hyperbolicity. *ACS Photonics*.

[51] Li L. (1997). New formulation of the Fourier modal method for crossed surface-relief gratings. *J. Opt. Soc. Am. A*.

[52] Li L. (1996). Formulation and comparison of two recursive matrix algorithm for modeling layered diffraction gratings. *J. Opt. Soc. Am. A*.

[53] Palik E. D. (1991). *Handbook of Optical Constants of Solids II*.

