# Supplementary material for: Prominently enhanced luminescence from a continuous monolayer of transition metal dichalcogenide on all-dielectric metasurfaces
Source: Nanophotonics. 2023 Dec 25;13(1):95–105. doi: 10.1515/nanoph-2023-0672 (PMC11501220; doi:10.1515/nanoph-2023-0672)
Supplement: Supplementary file 1 — Supplementary Material Details [file j_nanoph-2023-0672_suppl_001.pdf]

Supplementary Material: Prominently enhanced luminescence from a continuous monolayer of transition metal dichalcogenide on all-dielectric metasurfaces

Masanobu Iwanaga,[*,†] Xu Yang,[†,‡] Vasilios Karanikolas,[†,¶] Takashi Kuroda,[†] and Yoshiki Sakuma[†]

†National Institute for Materials Science (NIMS), 1-1 Namiki, Tsukuba 305-0044, Japan

‡Present address: Institute of Materials and Systems for Sustainability, Nagoya University, Nagoya 464-8601, Japan

¶Present address: Materials Modelling, Institute of Materials Science, Technical University of Darmstadt, 64287 Darmstadt, Germany

E-mail: iwanaga.masanobu@nims.go.jp

S1. Resonant Electromagnetic Fields

Figure S1 shows a set of electric- and magnetic-field intensity distributions in left and right panels, respectively, under the excitation condition at the reflectance peak E in Figure 2A. Scale bars indicate the intensities under condition that the incident intensity is set to unity. In both components, more than 100-fold field enhancement is observed. The magnetic field distribution, $|\mathbf{H}|^2$, exhibits a distribution quite similar to Figure 2D, strongly suggesting that the resonant mode is essentially the same to that appears in Figure 2D. The electric field distribution, $|\mathbf{E}|^2$, also shows a ditribution

qualitatively similar to Figure 2D.

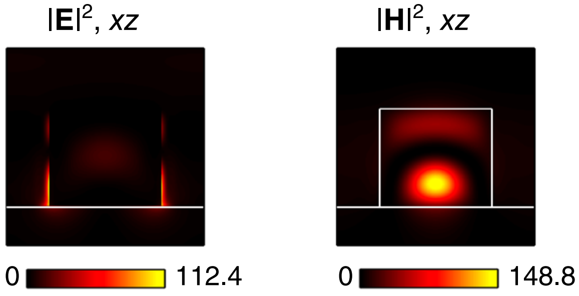

Figure S1: Electromagnetic field intensity distributions at the reflectance peak E in Figure 2A.

S2. Basic Properties of WS_2 Atomic Layer

Figure S2 shows optical properties of an as-grown WS_2 atomic layer in this study. They were measured at room temperature. Figure S2A presents Raman scattering spectrum with WS_2-mode indications of E^1_{2g} and A_{1g} and MoS_2-mode red arrows. Thus, the atomic layer is, strictly, $W_xMo_{1-x}S_2$. The ratio x is estimate to be $x \geq 0.9$ or less from the comparison with literature,[S1] indicating that W is dominant. Therefore, in Figure S2, we call the TMDC WS_2 for simplicity.

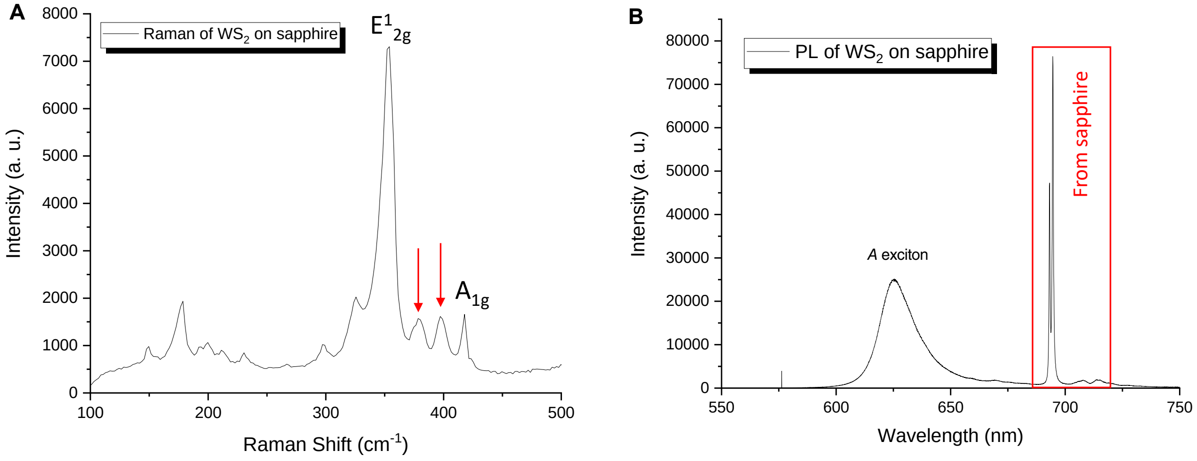

Figure S2: (A) Raman scattering and (B) PL spectra of an as-grown WS_2 atomic layer on a c-plane sapphire substrate. The spectra were measured at room temperature.

Figure S1B shows photoluminescence (PL) spectrum; a red box surrounds the PL coming from the ions in the sapphire substrate. The PL originating from the A exciton had a peak at 625 nm.

S3. Analysis of Confocal PL Images

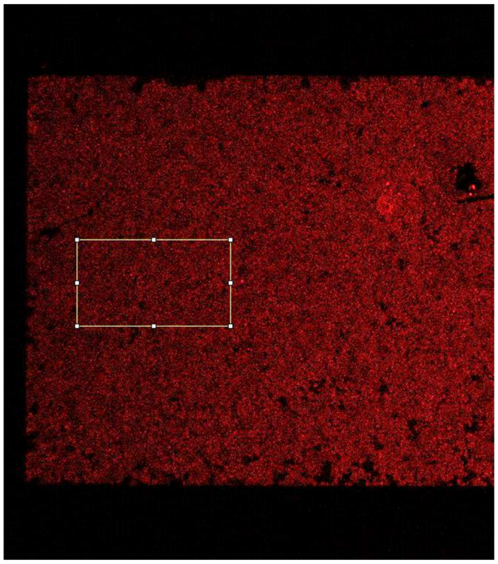

Figure S3: Analysis setting of confocal PL image. Box indicates the analyzed area for the photon-number distribution in Figure 3C. Note that the color of the image is pseudo-color and the original is gray-scale.

Figure S3 shows the confocal PL image, identical to Figure 3B, together with an analyzing box for the photon-number distribution (Figure 3C). The box contained 39644 pixels, being set to avoid rifts and holes as possible in the TMDC atomic layer. We note that the photon-number distribution was not affected significantly by the setting of the box. The confocal image is gray-scale and shown with a pseudo-color; the color is different from that in Figure 3B.

S4. On-Top Electric-Field Intensity

Figure S4 shows electric-field intensities in the optical configuration of Figure 2. The *xy* sections were set to be 0.5 nm above the top of the Si nanopellet. White circles indicate the position of Si nanopellet in the unitcell. Incident field was set to 1.0.

Figure S4A shows that the electric-field intensity at 532 nm (i.e., excitation wavelength) is enhanced by 5.3-fold at the maximum, and Figure S4B shows that at 608.9 nm, where the reflectance

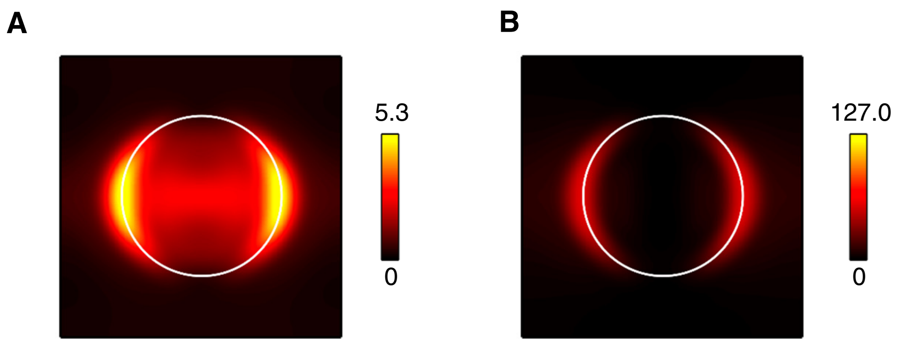

Figure S4: Electric-field intensity at 0.5 nm above the top of Si nanopellet: xy-section view. (A) 532.0 nm. (B) 608.9 nm. Incident intensity was set to 1.0.

takes a peak and the PL was largely enhanced (Figure 3A). In the latter, the maximum is 55.6 and the resonant field is quite stronger than the incident field.

S5. Purcell Effect: A Numerical Approach

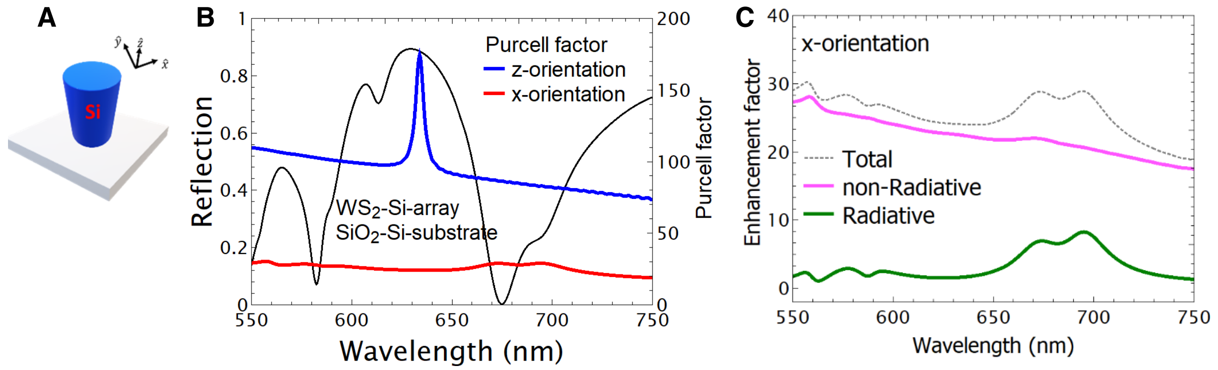

Figure S5: Theoretical evaluation of Purcell factors. (A) Coordinate configuration for the unitcell. (B) Total Purcell factors at x and z polarizations, shown with red and blue curves, respectively. The factors are plotted for the right axis. Normalized reflection spectrum is also shown with black curve. (C) Purcell (or enhancement) factors are shown in a decomposed manner into radiative and nonradiative parts. Green curve corresponds to the experimental condition.

Theoretical setting for Purcell-factor evaluation is described in the text (Methods section). Figure S5A shows the coordinate configuration. A two-level oscillator was set to be placed at the center of Si nanopellet above 2 nm from the top circular surface.

Figure S5B presents reflection spectra (black) plotted for the left axis and total Purcell factors

at x and z polarizations (red and blue, respectively) plotted for the right axis. The total factors contain both radiative and nonradiative components. The experimentally observed Purcell factor (or enhancement factor) corresponds to a x-polarized radiative component shown with a green curve in Figure S5C. The realistic factor takes values around 2 in 600–650 nm. Note that the total Purcell factor (dotted line) in Figure S5C is identical to that at x polarization (red) in Figure S5B.

S6. Transfer Procedure of TMDC Monolayer

When transferring the TMDC monolayer, we used polymethyl methacrylate (PMMA) film for protection. As described in the text (section 4.2), the PMMA was spin-coated on the as-grown TMDC film on the sapphire substrate. Immersing the substrate in water, the TMDC and PMMA film was peeled out together from the substrate. This peeled-out film was placed on the metasurface, which is schematically illustrated in Figure S6A (left). Via van der Waals force, the film and the metasurface substrate are absorbed. The PMMA protection film (light gray) was removed in acetone at 50 °C for 1 h (Figure S6A, center). Thus, we obtained the transferred TMDC film (yellow) on the metasurface (Figure S6A, right). Note that the metasurface under the TMDC film is omitted in the drawing. The sample in this study is schematically illustrated in a 3D view (Figure S6B); in reality, the atoms on the metasurface of Si-nanopellet array are much smaller than the Si nanopellets.

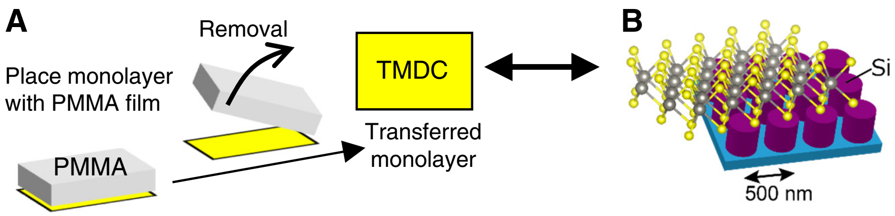

Figure S6: (A) TMDC monolayer (yellow) is protected using a PMMA film. The protection film is removed using organic solvent and transferred monolayer is prepared. (B) 3D-view illustration of TMDC-monolayer transferred on a metasurface of Si nanopellet array. Yellow spheres denote S atoms and gray ones W atoms.

References

(S1) X. An, W. Zhao, Y. Yu, *et.al.*, Resonance Raman scattering on graded-composition $W_xMo_{1-x}S_2$ alloy with tunable excitons. *Appl. Phys. Lett.*, vol. 120, no. 17, p. 172104, 2022.
